# Supplementary material for: AnVILWorkflow: A runnable workflow package for Cloud-implemented bioinformatics analysis pipelines
Source: F1000Res. 2024 Oct 21;13:1257. [Version 1] doi: 10.12688/f1000research.155449.1 (PMC11635187; doi:10.12688/f1000research.155449.1)
Supplement: Peer review history

# Reviewer report: Reviewer response for version 1

**DOI:** 10.5256/f1000research.170635.r342030

**Published:** 2024-12-11

**Authors:** Zachary S.L Foster

**Affiliations:** 1 USDA Agricultural Research Service, Corvallis, OR, USA

The authors describe an R package for submitting and interacting with pipelines hosted on the Genomic Data Science Analysis, Visualization, and Informatics Lab-space (AnVIL). The goal of this package is to make it easier for people primarily familiar with R to use AnVIL resources without learning other systems. This is primarily beneficial for those inexperienced in bioinformatics and that need to run large analyses using linux-based tools. The paper is succinct and written well. I appreciate the factual and unbiased tone of the writing. In particular, the lack of exaggeration of the significance of the work is refreshing.

 The tools itself seems fairly simple and is primarily consists of “quality of life” wrappers for the AnVIL R packages, which seem to more closely follow the implementation of the underlying API. AnVILWorkflow has about 10 commands, each 50-100 lines of R code. The code is well documented and seems tidy, but there are a few minor improvements that could be made, which I mention below. I installed the package from Bioconductor and the few function I tried seemed to work, but I did not run any analyses, since this would require an account or Terra and would cost money.

 Overall, this article is a nice and succinct description of a tool meant to make running analyses on Terra/AnVIL more user-friendly.

 Below are specific comments for each section.

**Introduction**

- Define acronym NHGRI
- “Enable to set up a Cloud environment with a single function call” sounds off. Maybe “Cloud environment initialization with a single function call.”
- Should “spent reports” be “spending reports” or “expense reports”?
- “However, the AnVIL package is not customized for workflow execution tasks.”: The source code for AnVILWorkflow::runWorkflow calls AnVIL::Terra()$createSubmission, so it seems that the AnVIL package does allow for workflow execution. Are you trying to say that it is not as convenient for workflow execution? Might want to rephrase this to make it more clear/accurate.

**Results**

- “Name of the Trimmomatic adaptor type”: Is this the right phrasing? Trimmomatic is the program used to remove adapters, but is not a type of adaptor. Perhaps something like “Sequencing adaptor type”?

**Discussion**

- “Despite not being inherently more costly than an in-house server, the pay-per-use structure requires careful planning and management.”: I like that this comparison is brought up. Cloud based analysis sounds very tempting at first, but I have used it for genomics data analysis and I am not convinced its a way to save money. In fact, I expect that in-house computing is actually cheaper in the long run. A powerful desktop can run the vast majority of bioinformatic workflows. Scaling and massive one-off analyses are the main benefits of cloud computing. Surprise costs are a big problem, especially to those that have little experience in commercial cloud computing, which is the target audience of this article. It might be a good idea to add a stronger warning about surprise costs. I also like that data egress fees were brought up earlier.
- “In conclusion, AnVILWorkflow proves most advantages for analyzing a bulk of samples on relatively simple workflows”: Is the “most advantages” phrased right? “most advantageous”? Also, the lack of exaggeration when describing the significance of the work is refreshing.

**Github.io documentation**

- The image on the home page does not load. Looks like a broken link.
- There are some errors that look like they came from chunks with echo = FALSE (e.g. “Error : ‘avworkspace_clone’ failed:” and “Error in mget(arg_names, environment): object ‘billingProjectName’ not found”). Perhaps the Rmd/Qmd needs to be fixed and rerun or make errors not printed?
- Typically there are installation instruction on the README, especially in combination with a quick start guide. I know there are instructions on Bioconductor, but the first place many people look is the Github repository README, so they should be there too. Consider adding the command to install the development version too using devtools::install_github().

**The source code**

- The code is documented well using roxygen2 and seems tidy Overall
- The jsonlite package is used for JSON parser, but a much faster package is available called RcppSimdJson. This should be a simple drop in replacement for the most part and will make json parsing 100s of times faster and more RAM efficient. However, this might not matter if only small JSON files are being parsed.
- There are some dependencies that could be replaced with base R code, which would make maintaining the code easier: dplyr, plyr, stringr, and tidyr. Some of these might not be worth the effort to replace, but stringr for example is only used in one place to convert something to title case. The only non-trivial thing dplyr and plyr are doing are table joins and the merge function can do this in base R. dplyr::filter could easily be replaced with standard R subsetting. I think the tidyverse packages are great for interactive use and data analysis scripts, but not ideal as dependencies since they are large, complex, and frequently break backwards compatibility. However, there is always a balance between dependencies and development time/effort, so this should be considered on a case-by-case basis.
- Consider adding a LICENSE file to the repository. I know its in the DESCRIPTION file, but that is specific to R, and LICENSE is the standard for most software and then Github will be able to read it and display the license, which is important information for many users.

Are the conclusions about the tool and its performance adequately supported by the findings presented in the article?

Yes

Is the rationale for developing the new software tool clearly explained?

Yes

Is the description of the software tool technically sound?

Yes

Are sufficient details of the code, methods and analysis (if applicable) provided to allow replication of the software development and its use by others?

Yes

Is sufficient information provided to allow interpretation of the expected output datasets and any results generated using the tool?

Partly

Reviewer Expertise:

I am primary a bioinformatic software developer with experience in pathogen diagnostics, metabarcoding, and developing pipelines. Of relevance to this article, I have developed R packages and pipelines that run on commercial cloud services.

I confirm that I have read this submission and believe that I have an appropriate level of expertise to confirm that it is of an acceptable scientific standard.

# Reviewer report: Reviewer response for version 1

**DOI:** 10.5256/f1000research.170635.r342032

**Published:** 2024-12-04

**Authors:** Fabricio Almeida-Silva

**Affiliations:** 1 VIB Center for Plant Systems Biology, Ghent University, Ghent, Belgium

In this manuscript, Oh et al present AnVILWorkflow, an R package that allows running and managing AnVIL workflows directly from an R session. This package is a valuable resource for the bioinformatics community, especially to novice bioinformaticians or researchers who are not familiar with the details of AnVIL. AnVILWorkflow has the potential of making AnVIL more widely used and known, simplifying the analysis of biological data and helping decelerate the unnecessary growth of lab-specific bioinformatics pipelines that do (roughly) the same thing. The manuscript is well written, and it nicely describes what AnVIL is, positive and negative aspects, and how to use AnVIL workflows in R. Overall, I believe the manuscript is in great shape in its current format. I have only a few minor points that I believe could help readers:

 (1) Authors mention some approaches to reduce costs, such as using call caching and pre-emptive instances. I believe it would be useful if authors could expand on that, giving clear examples (maybe using one of the three example workflows in the manuscript) on how such cost reductions can be achieved.

 (2) For people who have never used AnVIL before and want to consider using it after reading this paper, I think authors could add a few sentences in the Discussion describing how to set up AnVIL in a lab. For instance, since it works on a pay-per-use basis, should a lab have a single account or should each member of the lab have its own? How should accounts be managed to reduce redundancy and costs?

 (3) The whole idea of AnVIL seems very similar to Galaxy. I know the purpose of this manuscript is to describe AnVILWorkflow, not AnVIL itself, but maybe mentioning Galaxy in the Introduction and how AnVIL differs from Galaxy (highlighting advantages and disadvantages) would be useful to readers.

Are the conclusions about the tool and its performance adequately supported by the findings presented in the article?

Yes

Is the rationale for developing the new software tool clearly explained?

Yes

Is the description of the software tool technically sound?

Yes

Are sufficient details of the code, methods and analysis (if applicable) provided to allow replication of the software development and its use by others?

Yes

Is sufficient information provided to allow interpretation of the expected output datasets and any results generated using the tool?

Yes

Reviewer Expertise:

Bioinformatics

I confirm that I have read this submission and believe that I have an appropriate level of expertise to confirm that it is of an acceptable scientific standard.
